# Morphometric Trajectory Analysis for Occipital Condyle Screws

**DOI:** 10.1111/os.12700

**Published:** 2020-06-03

**Authors:** Yu‐kun Du, Si‐yuan Li, Wen‐jiu Yang, Xiang‐yang Wang, Yi‐fang Bi, Jun Dong, Hui Huang, Feng Gao, Gui‐zhi Li, Hua‐wei Wei, Jian‐kun Yang, Yong‐ming Xi

**Affiliations:** ^1^ Department of Spinal Surgery The Affiliated Hospital of Qingdao University Qingdao China; ^2^ Department of Spinal Surgery Shandong Provincial Third Hospital, Cheeloo College of Medicine, Shandong University Jinan China; ^3^ Department of Orthopaedics The Second Affiliated Hospital and Yuying Children's Hospital of Wenzhou Medical University Wenzhou China; ^4^ The Sanatorium of Qingdao Qingdao China; ^5^ Department of Spinal Surgery Shandong Provincial Hospital affiliated to Shandong University Jinan China; ^6^ Department of Orthopaedics Nanyang City Center Hospital; ^7^ Department of Spinal Surgery De Zhou People's Hospital Dezhou China; ^8^ Department of Spinal Surgery Zhoukou Xiehe Orthopedic Hospital Zhoukou China

**Keywords:** Morphometric trajectory analysis, Occipital condyle screw, Occipitocervical fusion

## Abstract

**Objectives:**

Occipitocervical fusion (OCF) is an effective treatment for instability of occipitocervical junction (OCJ). The occipital condyle screw serves as a novel surgical technique for occipitocervical fixation. However, the intraoperative procedures for the occipital condyle screw technique have relied on surgeons’ experience, so the pool of surgeons who are able to perform this surgery safely is limited. The present study aims to evaluate the feasibility and safety of the occipital condyle screw technique using human cadavers and to provide image anatomy for clinical application basis.

**Methods:**

The scientific study comprised 10 fresh‐frozen cadaveric specimens from the anatomy department of Qingdao University. Placement of the occipital condyle screws (3.5 mm diameter and 20.0 mm length) was performed in the 10 fresh‐frozen cadaveric specimens with intact occipitocervical junctions, respectively. Occipitocervical CT was performed for all specimens and the DICOM data was obtained. Occipitocervical CT three‐dimensional (3D) reconstruction was performed for the cadavers. Morphometric analysis was performed on the bilateral occipitocervical junction of 10 cadaveric specimens based on the 3D reconstruction CT images. Detailed morphometric measurements of the 20 occipital condyles screws were conducted including the average length of the screw trajectory, inside and upper tilting angles of screws, distance to the hypoglossal canal, and to the medial wall of occipital condyle.

**Results:**

Placement of the occipital condyle screws into the 20 occipital condyles of the 10 cadaveric specimens was performed successfully and the trajectory of implantation was satisfactory according to 3D CT reconstruction images, respectively. There was no obvious injury to the spinal cord, nerve root, and vertebral artery. The length of the bilateral screw trajectory was, respectively, 20.96 ± 0.91 mm (left) and 20.59 ± 0.77 mm (right) (*t* = 1.306, *P* > 0.05). The upper tilting angle of bilateral screws was, respectively, 11.24° ± 0.74° (left) and 11.11° ± 0.64° (right) (*t* = 0.681, *P* > 0.05). The inside tilting angle of bilateral screws was, respectively, 31.00° ± 1.32° (left) and 30.85° ± 1.27° (right) (*t* = 0.307, *P* > 0.05). The screw's distance to the bilateral hypoglossal canal was, respectively, 4.84 ± 0.54 mm (left) and 4.70 ± 0.54 mm (right) (*t* = 0.685, *P* > 0.05). The screw's distance to the medial wall of the bilateral occipital condyle was, respectively, 5.13 ± 0.77 mm (left) and 5.04 ± 0.71 mm (right) (*t* = 0.384, *P* > 0.05).

**Conclusion:**

The occipital condyle screw technique can serve as a feasible and safe treatment for instability of the occipitocervical junction with meticulous preoperative planning of the screw entry point and direction based on individual differences. Morphometric trajectory analysis is also an effective way to evaluate the surgical procedure.

## Introduction

The most flexible section of the cervical spine is the occipital‐C_1_–C_2_ complex, which is mainly responsible for flexion (21°), extension (3.5°), and axial rotation (23.3°–38.8° per side)^1^. The occipitocervical junction (OCJ) is composed of the facies articularis superior of massa lateralis atlantis and the occipital condyle (OC). Many important anatomic structures are adjacent to the occipital condyle: above it are the condyloid foramina and hypoglossal nerves, situated medially is the brain stem, posteriorly are the vertebral artery and the cervical nerve root and laterally are the emissary veins and the sigmoid sinus, while the retropharyngeal soft tissue is on the ventral side^2^. As a matter of fact, the hypoglossal canal that sits atop the occipital condyle is traditionally considered part of the occipital condyle. The mean distance from the hypoglossal canal to the inferior border of the occipital condyle is 11.5 mm^3^. The thickest part of the occipital bone is the external occipital protuberance, which is 9.7–15.8 mm thick^4^. The superior nuchal line directly faces the bilateral transverse sinus; the superior sagittal sinus passes downward from the calvaria along the median line and drains into the confluence of sinuses, which is opposite the inion. Anatomical study indicates that spine surgeons are presented a challenge because of the unique anatomic and kinematic relationships of this region^5^.

Various factors can result in occipitocervical instability, leading to local pain, spinal nerve compression symptoms, and even death, in a multitude of conditions including infection, trauma, tumor, and other degenerative conditions^6^. The stabilization of the OCJ not only can prevent compression of the nerve root but also rectify deformity of the upper cervical spine to reduce neck discomfort. Occipitocervical fusion (OCF) will cause substantial restrictions resulting in partial deficiency of the neck's range of motion because of the involvement of the occipital‐C_1_–C_2_ complex. Posterior internal fixation with instrumentation in OCF is still the treatment of choice for occipital cervical instability^7^. In 1927, the approach was first described by Foerster, who successfully achieved OCF for treatment of patients with OCJ instability using just a fibular strut graft^8^. Without internal fixation, initially, external immobilization and bed rest were required for an extended period of time. The fusion achievement, approximately 75% to 90%, was fair.

Despite improved surgical techniques, the halo immobilization was still essential because of the lack of internal fixation strength in the 1970s. Furthermore, the prolonged external immobilization could vastly affect the short‐term prognosis as well as the quality of life of postoperative patients^9^. The advent of internal fixation was the turning point for OCF. The plate/screw and rod/screw instruments could achieve re‐establishment of occipitocervical stability immediately and eliminate the need for external immobilization. The fusion achievement was almost 95% with the application of internal fixation. The placement of supplemental bone onlay graft after removing the cortical bone is essential for achieving bone fusion in the future^10^. Many advantages of screw‐based constructs are obvious, including efficient rigidity of fixation, high rate of fusion, and less involvement of motion segments than for any wire/cable fixation. Currently, there are various procedures for OCF, including use of occipital plates, hinged rods with an integrated occipital plate end, and eyelet connectors directed medially^11^. In cases of osteoporosis, when there is a thin occipital bone, or in the absence of occipital bone, the recently introduced OC screw is used. The surface of OC provides a satisfactory area for screw insertion to avoid postoperative complications such as dural sinus perforation.

Many studies have demonstrated that OC screws are a feasible option for treatment of occipitocervical instability, with no apparent neurovascular complications^12^. Recently, a biomechanical study indicated that OC screws and cervical screws are biomechanically equivalent in four physiological motions (flexion, extension, lateral bending, and axial rotation) to the standard occipital plate and cervical screw construct in terms of motion restriction and stiffness^13^. However, because of the high complexity inherent to adjacent neural and vascular structures, the recognition of aberrant vertebral artery distribution on preoperative imaging studies is also critical. Full knowledge of the precise angle and depth of OC screw insertion is necessary for surgeons. However, there are few reports in the literature on the applied anatomy of the adult occipital condyle, especially for OC fixation. In addition, few morphometric trajectory analyses have been undertaken to verify the accuracy of occipital condylar screws.

Therefore, we analyzed the anatomy of the OC in detail. The anatomic relationships between the OC screw and the vertebral artery, the medulla oblongata, and the foramen hypoglossal were also studied. CT‐based morphometric measurements of the craniovertebral junction for OC screw placement were performed, including screw insertion length, the upper tilting angle and inside tilting angles, and other parameters relating to the screws.

The aim of the present study was to: (i) enable surgeons to further study the complex anatomy of the occipital‐C_1_–C_2_ complex; (ii) allow surgeons to evaluate the safety and feasibility of the occipital condyle screw; and (iii) provide spine surgeons with a relevant theoretical basis for clinical application.

## Materials and Methods

### *Experimental Equipment*

Ten fresh‐frozen cadaveric specimens with intact OCJ were obtained from the department of anatomy at Qingdao University. Screws (diameter: 3.5 mm; length: 20.0 mm) and other surgical instruments were provided by Stryker (USA). We used a 64‐slice spiral CT machine manufactured by Siemens. This study was conducted in accordance with the Declaration of Helsinki and with approval from the Ethics Committee of Qingdao University.

### *General Information*

#### *Participants*

Ten cadaver specimens were enrolled in the study. They were all obtained from the department of anatomy, Qingdao University. The cadaver specimens all had intact OCJ. All the cadaver specimens had undergone no previous cervical surgery. Ten cadaver specimens had no obvious deformity or vertebrae fractures at OCJ.

#### *Interventions*

All surgical procedures were performed by three experienced surgeons. All specimens underwent thin‐slice CT scanning, respectively. According to the screw entry point and screw trajectory, based on the measurements of CT images, screws were inserted into the OC of specimens.

#### *Surgical Procedures*

All specimens were placed on the operation table, with the head in a cephalostat in the prone position. A nuchal midline incision was made and it was extended from the external occipital protuberance to the C_3_–C_4_ level. The skin and subcutaneous fascia were dissected in turn. Muscles were laterally retracted to expose the posterior arch of the atlas. The horizontal segment of the vertebral artery was identified by dissecting laterally in a subperiosteal fashion. Following the exposure of the occipital bone, C_1_ and C_2_, the posterior atlantooccipital membrane was then dissected laterally from the foramen magnum to the medial margin of the OC. The soft tissues around the screw entry points were dissected and removed using the operating scalpel and nerve dissector to identify the bony landmark. The placement of the screws was performed at the bilateral entry points according to the measurements.

#### *Comparisons*

**Fig. 1:**
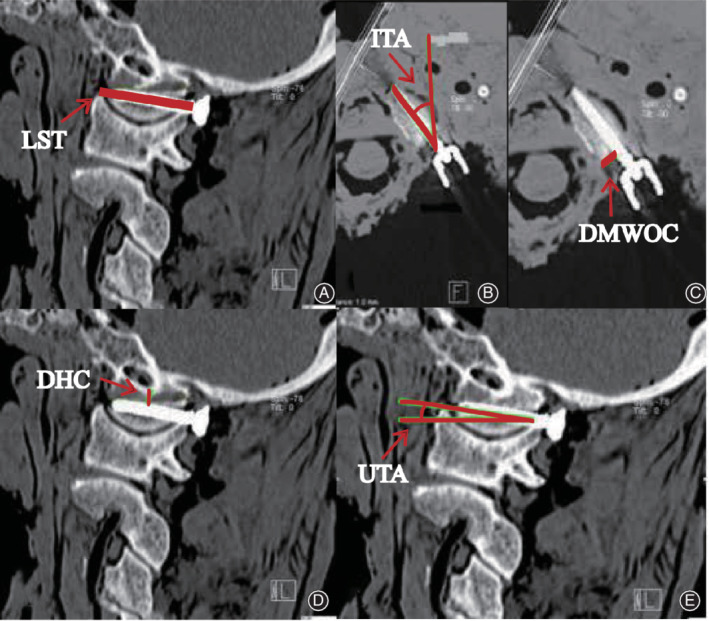
The measurements of (A) the length of screw trajectory (LST), (B) the inside tilting angle (ITA), (C) the distance to the medial wall of the OC (DMWOC), (D) the distance to hypoglossal canal (DHC) (D), and (E) the upper tilting angle (UTA). CT images show the different parameters for screw insertion.

A 64‐slice spiral CT scan with a slice thickness of 1 mm was performed. The 3D reconstruction images of the sagittal and axial OCJ views along the long axis of the OC were made by using a GE 3D CT image processing workstation (Fig. 1).

#### *Outcomes*

Bilateral placement of 20 OC screws was achieved. We, respectively, measured the bilateral 20 OC screws in the aspects of the length of screw trajectory (LST), the upper tilting angle (UTA), the inside tilting angle (ITA), the distance to hypoglossal canal (DHC), and the distance to the medial wall of the OC (DMWOC) of the OC screws. We also examined the relationships between the screws and the adjacent structures using the 3D CT images (Fig. 1).

### *Clinical Measurements of Occipitocervical Screws*

#### *Length of Screw Trajectory*

The LST was the length of the screw trajectory within the bone. It can be measured based on CT images to help surgeons understand appropriate depth of screw insertion during an operation (Fig. 1).

#### *Upper Tilting Angle*

The UTA was the head tilting angle of the screw insertion in the sagittal plane. The sagittal CT images can show the screw insertion angle to help surgeons identify the appropriate head titling angle of the screw insertion to avoid damage to vessels and nerve injury.

#### *Inside Tilting Angle*

The ITA is the inside‐and‐outside tilting angle of the screw insertion in the horizontal plane. To avoid damage to vessels and nerve injury, the ITA can be measured from horizontal CT images to help surgeons identify the inside‐and‐outside tilting angle of the screw insertion.

#### *Distance to Hypoglossal Canal*

The DHC was the inside distance between the occipital condyle screw and the hypoglossal canal in the sagittal plane. The hypoglossal canal is the passageway for the hypoglossal nerves. The DHC can be measured in the sagittal CT images.

#### *Distance to Medial Wall of the Occipital Condyle*

The DMWOC was the inside distance between the occipital condyle screw and the medial wall of the occipital condyle in the horizontal plane. The DMWOC can be measured in the horizontal CT images.

### *Statistical Analysis*

The data was analyzed using the software package SPSS 25.0 (IBM, USA). Statistical analysis was performed using paired samples *t*‐tests to analyze the LST, UTA, ITA, DHC, and DMWOC. The data were presented as mean ± SD. *P* < 0.05 was regarded as statistically significant.

## Results

### *Measurements of Length of Screw Trajectory*

The measurements of LST on both sides of the 10 cadavers were obtained based on sagittal CT images. The LST was 20.96 ± 0.91 mm on the left side and 20.59 ± 0.77 mm on right side. There was no statistically significant difference in the LST on either side (*t* = 1.306, *P* > 0.05).

### *Measurements of Upper Tilting Angle*

The measurements of UTA on both sides of the 10 cadavers were obtained. The UTA was 11.24° ± 0.74° on the left side and 11.11° ± 0.64° on the right side. No statistically significant differences in UTA were shown for either side (*t* = 0.681, *P* > 0.05).

### *Measurements of Inside Tilting Angle*

The measurements of ITA on both sides of the 10 cadavers were obtained. The ITA was 31.00° ± 1.32° on the left side and 30.85° ± 1.27° on the right side. There was no statistically significant difference in the ITA on either side (*t* = 0.307, *P* > 0.05).

### *Measurements of Distance to Hypoglossal Canal*

The measurements of DHC on both sides of the 10 cadavers were obtained. The DHC was 4.84 ± 0.54 mm on the left side and 4.70 ± 0.54 mm on the right side. No statistically significant differences in DHC were shown for either side (*t* = 0.685, *P* > 0.05).

### *Measurements of Distance to the Medial Wall of the Occipital Condyle*

**Fig. 2:**
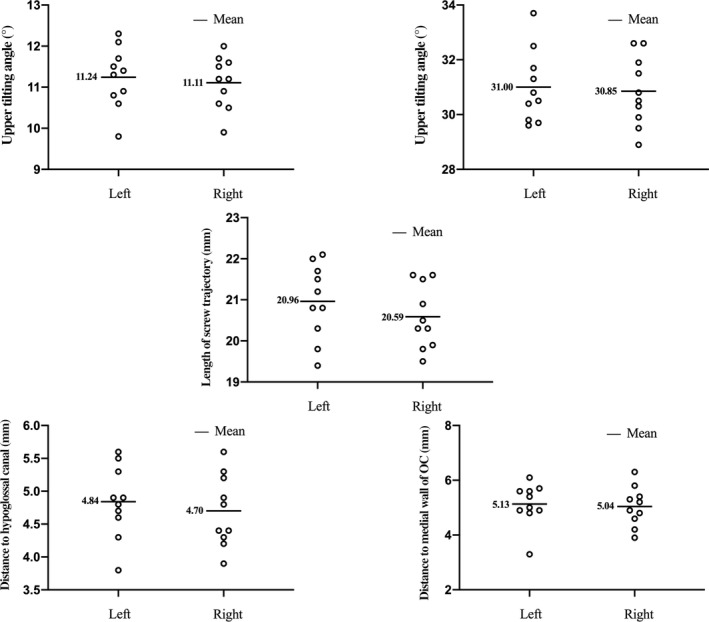
Graphs show that there is no significantly bilateral difference of the LST (middle), UTA (top left), ITA (top right), DHC (bottom left), and DMWOC (bottom right).

The measurements of DMWOC were obtained for both sides of the 10 cadavers. The DMWOC was 5.13 ± 0.77 mm on the left side and 5.04 ± 0.71 mm on the right side. There was no statistically significant difference in DMWOC on either side (*t* = 0.384, *P* > 0.05) (Fig. 2, Table 1).

**TABLE 1:** The measurements of LST, UTA, ITA, DHC, and DMWOC (mean ± SD)

Sides	LST (mm)	UTA (°)	ITA (°)	DHC (mm)	DMWOC (mm)
Left side	20.96 ± 0.91	11.24 ± 0.74	31.00 ± 1.32	4.84 ± 0.54	5.13 ± 0.77
Right side	20.59 ± 0.77	11.11 ± 0.64	30.85 ± 1.27	4.70 ± 0.54	5.04 ± 0.71
*t*‐value	1.306	0.681	0.307	0.685	0.384
*P‐*value	>0.05	>0.05	>0.05	>0.05	>0.05

## Discussion

Numerous factors can lead to instability at the OCJ, including trauma, inflammation, neoplasm, and congenital and iatrogenic causes. OCF is an effective treatment for OCJ instability. However, meticulous preoperative assessment is needed because of the unique anatomical location.

### *Occipitocervical Fusion for Occipitocervical Junction*

Several OCF methods have been applied since Foerster first described the approach in 1927^8^. Early OCF techniques mainly used tight wires to fix the bone grafts. However, using steel wires and tight wires, there is the risk of injuring the nerves. In 1986, Ransford *et al*.^14^ reported an OCF technique involving pre‐molded U‐shaped metal bars that fitted the OCJ configuration. The metal bar was fixed to the occipital bone by a steel wire passing through a drill hole in the occipital bone. However, this technique is not widely used because of the difficulty for the bars to integrate with bone and prolonged external fixation.

With the development of lateral mass screw techniques, plate systems began to be widely used in occipitocervical fusion in the early of 1990s. A long‐term follow‐up study carried out by Sasso *et al*.^15^ demonstrated that the backside plate and the lateral mass screw internal fixation technique achieved a high rate of fusion. However, severe problems remained, and it was impossible for the screws to be placed into an optimal location if the screw holes did not fit the patient's anatomy. It was not appropriate for bone grafts because of the restricted placement area. In addition, the plate cannot sustain compression or distraction. Therefore, plate systems were gradually replaced by modular systems in the late 1990s. According to Grob *et al*.^16^, the current available internal fixation systems often fail to solve the abovementioned problems.

### *Challenges for Occipitocervical Fusion*

The most common surgery requires integrity of the posterior part of the occipital bone, and the thickness and strength can impact bone fusion. The limitations of the occipital bone sclerosis associated with lateral occipital fixation pose the biggest problem for the occipitocervical fusion^17^. In fact, the occiput area available for the fixation of screws is limited. The available area for fixation could be restricted further and does not accommodate instrumentation during a suboccipital craniectomy for decompression. In some cases, the sclerotin of the occipital bone is very thin, including Chairi's deformity, which makes it impossible to achieve a firm fixation. In addition, lateral occipital fixation requires a very low incision to prevent scalp injury. The slope of the occipital bone and its angle with the cervical spine can also lead to difficulties with screw insertion^18^. A series of problems arise because of these limitations, including screw loosening, pullout, and breakage.

### *Occipital Condyle Screw Technique*

Uribe *et al*.^13^ described the surgical exposure procedures for applying OC screws on cadavers. According to Uribe *et al*., to avoid vertebral arterial injury, fully revealing the vertebral arteries should be done extremely carefully after exposing the atlantooccipital joint. Therefore, adequate skill is necessary to avoid serious postoperative complications. They made a standard posterior median incision from the external occipital protuberance to the spinous process of C_3_ or C_4_. To reveal the horizontal segments of the vertebral artery, dissection along the bilateral periosteum was performed after exposing the arcus posterior atlantis. Full exposure of the occipital bone,

C_1_ and C_2_ was achieved and then the membrane of C_1_ was dissected from the foramen magnum to the occipital condyle using a small sized curet. The dissection was then performed outwards from the foramen condyloideum to the emissary vein along the bone surface to prevent injuring the vertebral artery. The entry point was located on the junction of the occipital condyle and the occipital bone. The angle of the screw trajectory from medial to lateral and the tilt angle to the head were 12°–22° and 5°, respectively. The length of the screw trajectory was 20–24 mm. The author found that the occurrence of complications can be reduced and screwing safety can be significantly improved by certain careful anatomical operations. Cadaveric studies, aiming to evaluate the feasibility of occipital condyle screws, can verify the safe placement for screw insertion on the basis of thorough knowledge of the OC junction anatomy.

Naderi *et al*.^19^ performed morphological analysis using bony specimens of OC. The OC is important in linking the skull and the atlas. The atlantooccipital joint is composed of the superior articular surface of the massa lateralis atlantis and the occipital condyle. They converge ventrally with a mean sagittal angle of approximately 30° (10 to 54°). The upper part of the condyle is the hypoglossal canal. There are some adjacent important structures, including the jugular tubercle, the jugular foramen, the occipital condyle, and the sigmoid sinus. Wen *et al*.^20^ described the suboccipital triangle and all the neurovascular structures related to screw trajectory on this level. The occipital condyle‐type classification was reported. Ozer *et al*.^21^ suggested that an oval‐like type was the most common type, with low risk for the fixation resonance. Frankel *et al*.^22^ and Le *et al*.^23^ measured the occipital condyle of normal adults using CT images and revealed that: the average height, width, and length of the occipital condyle were close to 11 mm, 10 mm, and 22 mm, respectively.

In our study, the 20 OC were all successfully inserted with the screws, and no spinal cord, nerve root, vertebral artery or hypoglossal nerve was injured. We measured the LST, UTA, ITA, DHC, and DMWOC of the OC screws, and all the results were satisfactory. We used 24‐mm multi‐axis screws for insertion and the screwing entry points were 4–5 mm to the lateral side of the interior edge of the OC; namely, at the junction of the OC and the occipital bone. The DHC and the DMWOC, or the insertion points at the condylar process, were approximately 5 mm. The ideal OC screw channel should pass through the cross‐sectional center of the OC and be parallel to the long axis of the OC, but the screwing angle and an suitable screwing point cannot be achieved in actual operations^24^, ^25^.

In our study, all 20 cases had differences in the screw angles and entry points. However, the 3D reconstruction showed that the placed screws maintained adequate distances from the surrounding structures, so we believe that screws can be safely inserted with no damage caused to surrounding structures if the differences in the UTA and ITA are kept within 2°, and the screw entry points are kept 3–6 mm away from the lateral side of the posterior OC edge. Due to differences among races, the OC vary largely, but studies that anatomically measure the Chinese occipital condyles are limited.

We studied the 3D anatomies of the OC screws in these cadaver specimens and the relationships between OC screws and surrounding tissue structures, and further confirmed that OC screwing with a 30° interior inclination angle and a 10° upper inclination angle is also applicable for Chinese people, and will not damage the peripheral nerve and vascular structures. The screws can maintain a safe distance from the surrounding important structures: 20–24‐mm length screws are safe for use in Chinese and Asian people. We believe that preoperative 3D CT reconstruction can better and more safely guide the screwing procedure based on measuring the OC anatomical morphologies in the reconstructed images, thus improving the screwing safety. As an important structure at the occipitocervical site, the atlantoaxial joint bears 40%–50% of the activity of the cervical spine; the rest is borne by the lower cervical spine. Therefore, appropriately applying the OC screwing technology can maximally retain the cervical activity and achieve better therapeutic effects.

Of course, there are still issues with OC screws, including lack of uniform standards, and no specific screw can be selected for the OC. The bones of specimens were not the first option for biomechanical measurements. If OC screws were to be widely used in clinical practice, further biomechanical measurements should be performed to improve the technology. In addition, minimally invasive and precision insertion of OC screws could be performed using a navigation system and robots in the future.

Due to unfamiliarity with the anatomical structures around the OC and the serious potential complications caused during the screwing process, this technology is limited in clinical practice. However, the anatomical measurements of the OC have confirmed that it could be a more stable and reliable internal fixation method than traditional OCF methods. Therefore, the OC screw technique could improve on or supplement traditional OCF methods.
